# The *Alisma* and *Rhizoma* decoction abates nonalcoholic steatohepatitis-associated liver injuries in mice by modulating oxidative stress and autophagy

**DOI:** 10.1186/s12906-019-2488-6

**Published:** 2019-04-29

**Authors:** Lijun Xu, Menghui Jing, Lijuan Yang, Lei Jin, Peiqiang Gong, Juan Lu, Hui Lin, Jinping Wang, Qin Cao, Yuanye Jiang

**Affiliations:** 1 Department of Gastroenterology, Putuo Hospital, Shanghai University of Traditional Chinese Medicine, Shanghai, 200062 China; 2 Department of Gastroenterology, Shanghai General Hospital/First People’s Hospital, School of Medicine, Shanghai Jiao Tong University, Shanghai, 201620 China; 3 Department of Gastroenterology, Shaoxing Hospital of Traditional Chinese Medicine, Shaoxing, 312000 China

**Keywords:** *Alisma orientale*, *Rhizoma atractylodis macrocephalae*, Non-alcoholic fatty liver disease, Liver inflammation, Immunoblotting assays

## Abstract

**Background:**

To investigate the effects of the *Alisma* and *Rhizoma* decoction on nonalcoholic steatohepatitis (NASH) and to further shed light on the underlying mechanisms of the actions of the *Alisma* and *Rhizoma* decoction.

**Methods:**

Plasma alanine aminotransferase (ALT) content was determined and liver inflammation and fibrosis were evaluated. Intrahepatocellular malondialdehyde and superoxide dismutase contents were determined using commercially available kits Furthermore, α-SMA expression in liver tissues was examined by immunohistochemistry and LC3-II was detected by immunoblotting assays.

**Results:**

Mice receiving the *Alisma* and *Rhizoma* decoction by gastric lavage had significantly lower plasma ALT content and markedly higher hepatic superoxide dismutase activity than mice receiving the methionine-choline deficient (MCD) diet. Furthermore, the decoction aborted MCD-induced increase in liver malondialdehyde content. Immunohistochemistry showed that the decoction suppressed hepatic α-SMA expression. Our transmission electronic microscopy revealed that the decoction markedly reduced the number of autophagosomes and immunoblotting assays showed that the decoction caused a dose-dependent decrease in LC3-II in hepatic tissues.

**Conclusion:**

The *Alisma* and *Rhizoma* decoction lessens NASH-associated liver injuries by modulating oxidative stress and autophagy in hepatocytes of mice fed with MCD.

**Electronic supplementary material:**

The online version of this article (10.1186/s12906-019-2488-6) contains supplementary material, which is available to authorized users.

## Background

Non-alcoholic fatty liver disease (NAFLD) represents a broad spectrum of liver diseases ranging from simple steatosis to steatohepatitis through to fibrosis and cirrhosis [1] and is characterized by excessive lipid accumulation in the liver without chronic alcohol consumption. The global prevalence of NAFLD is 20–30%, affecting one billion persons worldwide [2]. The prevalence of NAFLD has been steadily increasing in China and has reached approximately 15% in economically developed metropolitan areas [3]. Non-alcoholic steatohepatitis (NASH) is a critical step in the progression of NAFLD and is marked by fatty degeneration of hepatocytes, necrotic inflammation and/or fibrosis and may further progress to hepatic fibrosis and even liver cancer. Liver fibrosis is a common entity underlying various chronic liver diseases and recently it has been shown development of liver fibrosis is a dynamic process and reversible under certain conditions [4].

Currently, the mechanisms underlying NASH liver fibrosis has not been fully elucidated and no effective treatment is available clinically. It has been recently reported that autophagy is implicated in the development and progression of hepatic fibrosis [5–7]. The autophagy flux increased when primary mouse hepatic stellate cells (HSC) became activated, which were suppressed by autophagy inhibitor afilomycin A1 [8]. Furthermore, autophagy-stimulated loss of lipid droplets was shown to modulate HSC activation [9]. These findings together suggest that autophagy may participate in the progression of fibrosis via modulation of HSC activation.

*Alisma orientale* (Sam.) Juzep. (Alismataceae) is a traditional medicinal herb [10] that has been used in China for treating a variety of conditions including hypertension, hyperlipidemia, Meniere’s disease and other conditions and *A. orientale* and its constituents have been shown to possess a broad spectrum of biological activities including anti-atherosclerotic activity, immunomodulation, and hepatoprotection [11–13]. Hong et al. showed that *A. orientale* methanolic extract improved the plasma lipid profile and alleviated hepatic steatosis in rats with high fat diet-induced NAFLD [10]. Jang et al. demonstrated that *A. orientale* attenuated hepatic steatosis of mice with high fat diet-induced obesity by suppressing endoplasmic reticulum (ER) stress. In vitro evidence also showed that *A. orientale* extract inhibited lipogenesis and lipoapoptosis of non-esterified fatty acid-treated HepG2 cells [14]. Alisol B 23-acetate, a natural triterpenoid isolated from Rhizoma Alismatis, the dried rhizome of *A. orientale*, was found to protect against non-alcoholic steatohepatitis in mice by activating the farnesoid X receptor [15]. *Rhizoma atractylodis macrocephalae* has long been widely used in East Asia as a traditional herbal medicine and constituents from *R. atractylodis macrocephalae* have been shown to possess anti-inflammatory activities in mice [16]. Fermented *R. atractylodis macrocephalae* was found to improve the lipid profile of rats with high fat diet-induced obesity [17].

The ancient Chinese medical scripture “the Inner Canon of the Yellow Emperor” mentions the use of the *Alisma* and *Rhizoma* decoction with ten equal parts of *A. orientale* and *R. atractylodis macrocephalae* for treating “alcoholic fever”, which manifests as lethargy and fever. Currently, the *Alisma* and *Rhizoma* decoction is widely used for the treatment of hyperlipidemia, fatty liver disease and Meniere’s syndrome [18–20]. Our previous clinical study found that compared to Western drugs, the *Alisma* and *Rhizoma* decoction alleviated liver injury and improved the lipid profile of NAFLD patients, suggesting that the decoction exerts hepatoprotective effects in NAFLD patients. However, the mechanisms whereby the *Alisma* and *Rhizoma* decoction exerts its myriad effects have not been elucidated.

We speculated that the *Alisma* and *Rhizoma* decoction could alleviate NASH-induced liver injuries via modulation of autophagy. In the current study, we established a mouse NASH model by the methionine-choline deficient (MCD) [21, 22] diet and investigated the effects of the *Alisma* and *Rhizoma* decoction on NASH mice and further shed light on the underlying mechanisms.

## Methods

### Animals

The study protocol was approved by the Experimental Animal Ethical Committee, Putuo Hospital, Shanghai University of Traditional Chinese Medicine. Animal study was carried out in strict accordance with the established institutional guidelines and the NIH guidelines on the use of experimental animals.

Thirty-six 8-week old male C57BL/6 mice weighing 20 ± 3.5 g were purchased from the Experimental Animal Center at the Putuo District Central Hospital, Shanghai University of Traditional Chinese Medicine. The animals were housed at a constant temperature (20–22 °C) at 50–70% humidity with a 12 h light/ dark photoperiod. Mice were provided with regular laboratory chow and water ad libitum and were allowed 1 week to accommodate.

### Treatments

*A. orientale* granules and *R. atractylodis macrocephalae* granules were identified and authenticated by the Traditional Chinese Medicine Pharmacy of Putuo District Central Hospital. The specimen voucher number is A1500730 for *A. orientale* granules and A1501391 for *R. atractylodis macrocephalae* granules. The stock was prepared at a ratio of 5 (*A. orientale*):2 (*R. atractylodis macrocephalae*) for gastric lavage. The mice were randomized to receive the regular diet and 2 mL distilled water (group I, *n* = 12), the MCD (Trophic Animal Feed High-Tech Co., Nantong, China) and 2 mL distilled water (group II, n = 12), or MCD and 2 mL the *Alisma* and *Rhizoma* decoction (4.31 g/kg) (group III, n = 12) by gastric lavage for 12 weeks.

### Biochemical determinations

Mice were anesthetized with 2% sodium pentobarbital (75 mg/kg) intraperitoneally. Venous blood was obtained via the orbital vein and centrifuged at 3000 rpm for 10 min. The animals were euthanized by cervical dislocation. Liver tissues were obtained via abdominal dissection. The above information is incorporated in the revised manuscript. The supernatant was saved and analyzed using a Vitros350 Automatic Biochemical Analyzer (Johnson & Johnson) for plasma alanine aminotransferase (ALT) content.

#### Hematoxylin and eosin (H&E) staining

At the end of 12 week treatment by gastric lavage, the animals were sacrificed by cervical dislocation and liver tissues were obtained via abdominal dissection and fixed in 4% paraformaldehyde and paraffin embedded. Serial sections were sliced at a thickness of 5 μm (Lecia, Wetzlar, Germany). Tissue sections were stained with H&E using the standard protocol. Briefly, the sections were stained in hematoxylin for 10 min, followed by staining in 1% eosin solution for 3 min, and subsequently washed with distilled water. The slides were then observed under a light microscope (BX51, Olympus Co.) for fatty changes of the liver, hepatic inflammation and fibrosis. NAFLD activity score (NAS) (ranging 0–8) was assessed as follows: 1) a score of 0 represented < 5% fatty degeneration of liver cells, 1 represented 5–33%, 2 34–66%, and 3 represented > 66% fatty liver degeneration; 2) a score of 0 represented lack of lobular inflammation (number of necrotic foci under 20× microscope),1 indicated the number of necrotic foci < 2; 2 indicated 2–4 necrotic foci, and 3 indicated > 4 necrotic foci; (3) a score of 0 and 1 represented no or scant balloon like changes of hepatocytes, and 2 indicted more balloon like changes. Each subscore was added and the total mean score was reported. NASH was excluded if NAS was < 3 and NASH was diagnosed if NAS was > 4. Liver inflammation and fibrosis were determined as previously described [23]: G0 indicates no inflammation; G1 mild inflammation; G2 moderate inflammation; G3 moderate-to-severe inflammation; G4 severe inflammation; S0 represents no fibrosis; S1 portal fibrosis without septa; S2 star-shaped portal fibrosis, with minimal septa; S3 portal fibrosis with numerous septa, but no pseudo lobe formation; S4 portal fibrosis with numerous septa and pseudo lobe formation.

### Immunohistochemistry

Immunohistochemistry was performed using the universal two-step immunohistochemical method. The expression of α-SMA in liver tissues was examined was detected using rabbit monoclonal antibody against α-SMA (catalog # sc-53,142; dilutions 1:200; Santa Cruz Biotechnology, Santa Cruz, CA). PBS was added instead of the primary antibody in the negative control.

### Transmission electron microscopy

Liver tissue blocks were postfixed in 2.5% glutaraldehyde for 2 h at room temperature, and the tissue was embedded in Epon 812. Ultrathin sections (50–80 nm) were cut with an ultramicrotome (Leica RM 2165, Leica Ultracut UCT, Germany) and stained with uranyl acetate and lead citrate. Observations and photomicrographs were made with a transmission electron microscope (TEM; JEOL JEM-2100, Japan) operated at 60 kV.

### Determination of malondialdehyde and superoxide dismutase contents

Homogenate of hepatic tissues was prepared as previously described [24, 25]. The tissue homogenate was centrifuged, and the supernatant was collected and stored at − 84 °C. Malondialdehyde and superoxide dismutase contents were determined using commercially available kits (Nanjing Jiancheng Bioengineering Institute, Nanjing, China) as instructed by the manufacturer. Measurements were done at least three times independently and averaged.

### Western blotting assays

Liver tissues were lyzed using RIPA lysis buffer containing a cocktail of protease inhibitors and phosphorylase inhibitors (Roche). Protein content in the lysates was determined by the BCA method. Samples were resolved by 8% SDS PAGE. Nonspecific binding was blocked with 5% bovine serum albumin. Anti-LC3-IIantibody (catalog # sc-292,354; dilutions 1:3000; Santa Cruz Biotechnology) and HRP-conjugated β-actin (catalog # HRP-60008; dilutions 1:1000; Proteintech Group, Wuhan, China). Membranes were visualized by using an ECL Plus Western Blotting Detection System (GE Healthcare, USA). Protein densitometry was performed using Image-ProPlus 6.0 image analyzing software (Media Cybe-netics Co.). LC3-II expression was normalized against β-actin.

### Statistical analysis

Data were expressed as mean ± standard deviations and analyzed using the SPSS23.0 statistical analysis software (SPSS Inc., Chicago, IL, USA). Comparison of multiple groups was done by ANOVA, and comparison between two groups used Student’s *t-*test. *P <* 0.05 was considered statistically significant.

## Results

### The *Alisma* and *Rhizoma* decoction lessens NASH-like changes in mice

**Fig. 1:**
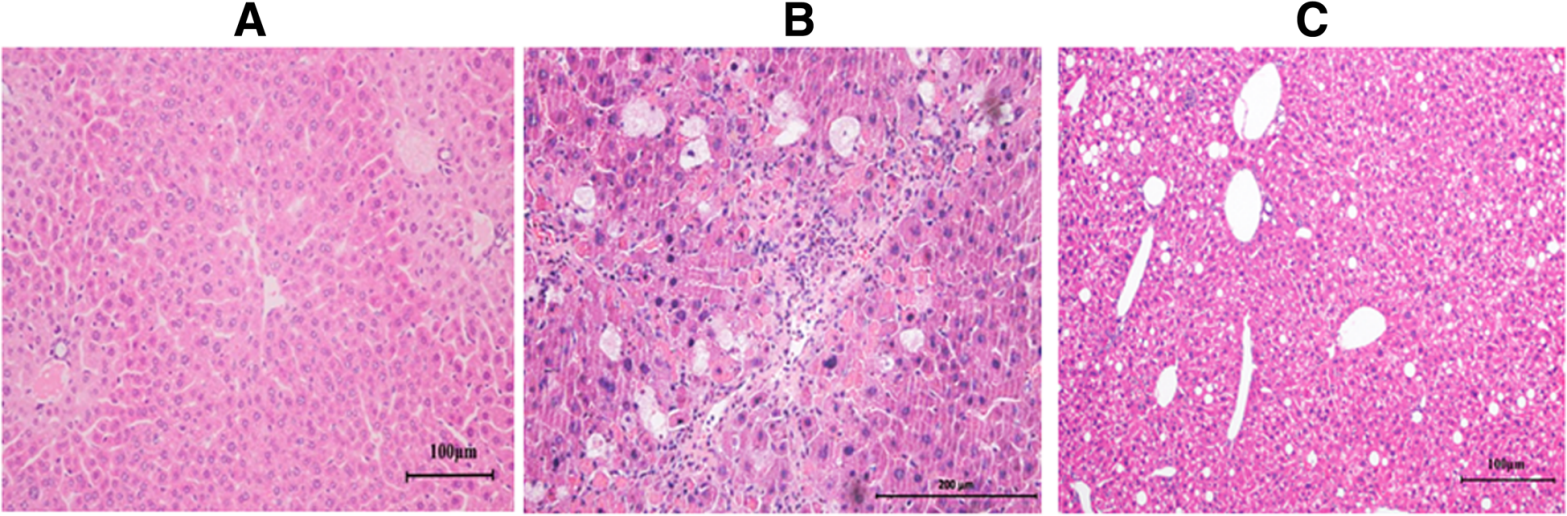
The *Alisma* and *Rhizoma* decoction lessens NASH-like changes in mice. Mice were randomized to receive the regular diet and 2 mL distilled water (**a**), the methionine and choline diet (MCD) and 2 mL distilled water (**b**), or the MCD and 2 mL *Alisma* and *Rhizoma* decoction (4.31 g/kg) (**c**) by gastric lavage for 12 weeks. H&E stained mouse liver tissue sections were examined for fatty changes of the liver, hepatic inflammation and fibrosis under a light microscope as described in Materials and methods. Magnification, 100 ×

We first examined the effect of the *Alisma* and *Rhizoma* decoction on MCD-induced NASH-like changes in mice. Histological examination showed that group I had normal liver architecture with intact hepatic lobules and with no fatty changes in hepatocytes (S0) (Fig. 1a). Group II showed indistinct lobule border, narrowed sinusoidal space and periportal fibrosis with septa (S2 and S3) (Fig. 1b). Massive punctate necrosis and balloon-like changes were observed, indicating that MCD induced NASH-like changes in these mice. By contrast, group III showed virtually no portal fibrosis and no septa (S1) and less severe balloon-like changes (Fig. 1c), suggesting that the *Alisma* and *Rhizoma* decoction attenuated MCD-induced NASH-like changes in mice.

### The *Alisma* and *Rhizoma* decoction attenuates MCD-induced liver injuries

**Fig. 2:**
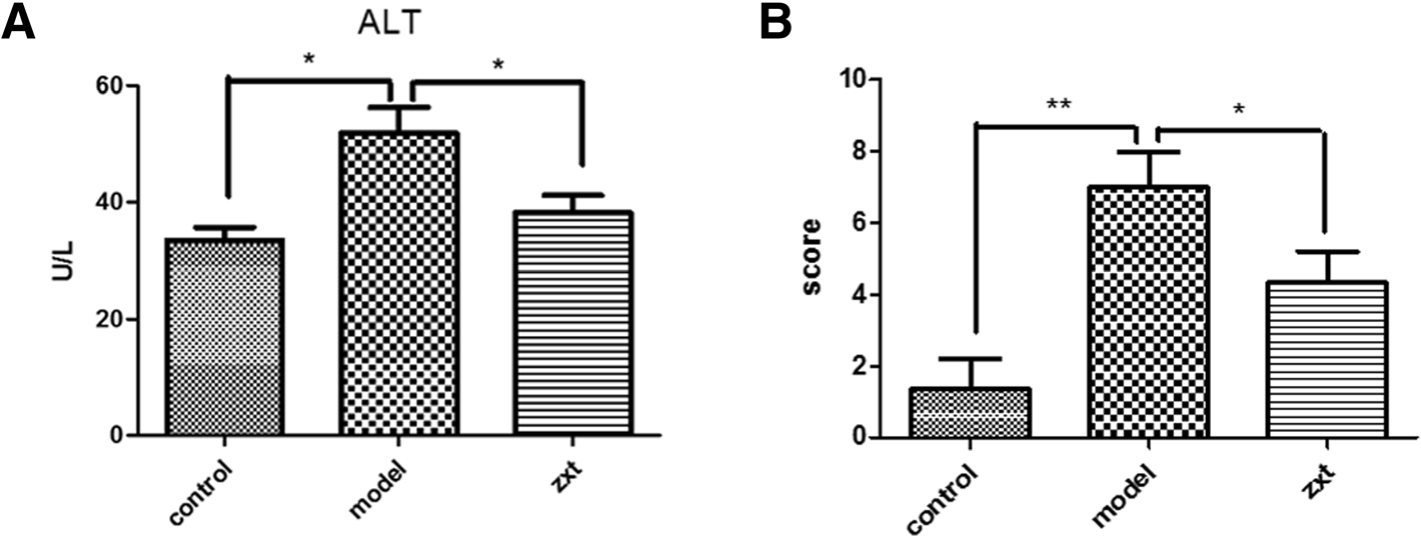
The *Alisma* and *Rhizoma* decoction attenuates MCD-induced liver injuries. Mice were randomized to receive the regular diet and 2 mL distilled water (group I), the methionine and choline diet (MCD) and 2 mL distilled water (group II), or the MCD and 2 mL *Alisma* and *Rhizoma* decoction (4.31 g/kg) (group III) by gastric lavage. **a** Serum ALT content in each group. **b** NAS scores in each group. *P < 0.05, **P < 0.01

We were interested in whether improvement by the *Alisma* and *Rhizoma* decoction in liver morphology in mice with MCD-induced NASH-like changes was associated with amelioration in hepatic function. The plasma ALT content in group I was33.5 ± 3.9 IU/mL. The plasma ALT content (51.8 ± 7.8 IU/mL) in group II was significantly higher than that of group I. This rise in plasma ALT content, however, was significantly attenuated in mice fed with the *Alisma* and *Rhizoma* decoction (38.2 ± 5.2 IU/mL) (*P <* 0.01) (Fig. 2a).

In addition, the NAS score in group II (7.00 ± 1.73 was significantly higher than that of group I (1.33 ± 1.53) (*P <* 0.05). The *Alisma* and *Rhizoma* decoction markedly attenuated MCD induced increase in NAS score (*P <* 0.05) (Fig. 2b). The above findings suggested that the *Alisma* and *Rhizoma* decoction attenuates MCD-induced liver injuries.

### The *Alisma* and *Rhizoma* decoction reduces oxidative stress in MCD-induced NASH

**Fig. 3:**
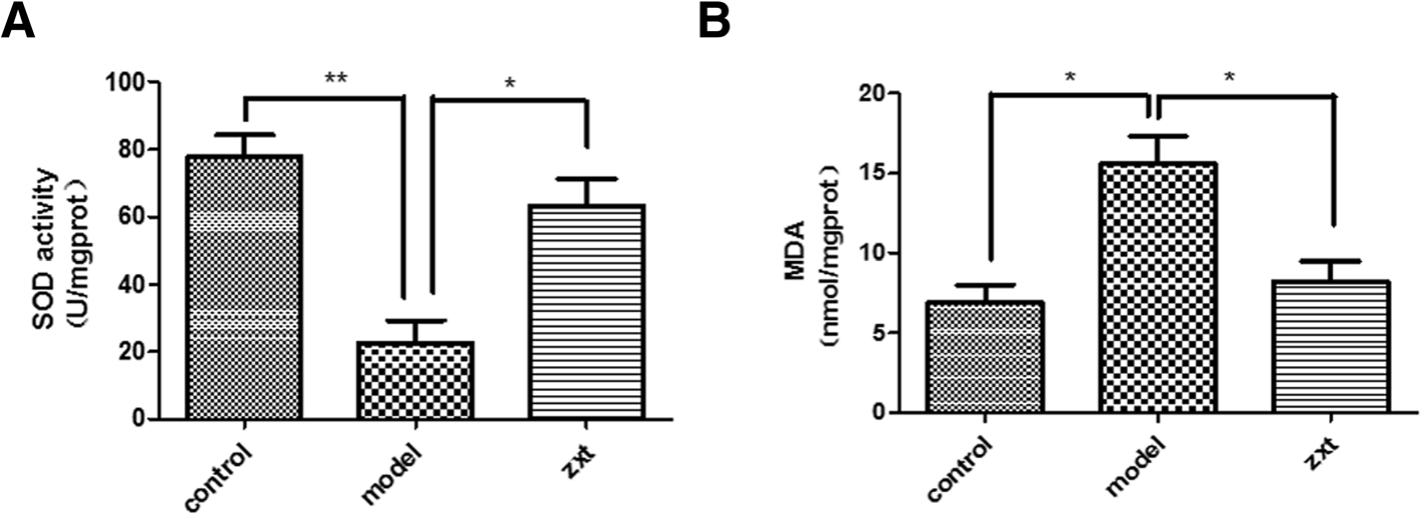
The *Alisma* and *Rhizoma* decoction reduces oxidative stress in MCD-induced NASH. Mice were treated as detailed in Fig. 2. The SOD activity (**a**) and MDA content (**b**) of the liver homogenate were measured as described in Methods. *P < 0.05, **P < 0.01

It has been reported that the aqueous extract of *A. orientale* protected against long-chain saturated fatty acid-induced cellular injury by attenuating oxidative stress [26]. We also found that the SOD activity of the liver homogenate was 22.67 ± 11.45 U/mg protein in group II, which was significantly lower than that of group I (77.76 ± 11.33 U/mg protein) (*P <* 0.01). This decrease, however, was markedly abated by treatment with the *Alisma* and *Rhizoma* decoction (63.14 ± 14.08 U/mg protein) (*P <* 0.05) (Fig. 3a). Furthermore, the MDA content of the liver homogenate was 6.93 ± 1.88 mmol/mg protein in group I. MCD caused a significant increase in liver MDA content (15.65 ± 2.92 mmol/mg) (*P <* 0.05 versus group I). This increase, however, was aborted by the *Alisma* and *Rhizoma* decoction (8.25 ± 2.16 mmol/mg) (*P <* 0.05 versus group II) (Fig. 3b).

### The *Alisma* and *Rhizoma* decoction suppresses α-SMA expression in MCD-induced NASH

**Fig. 4:**
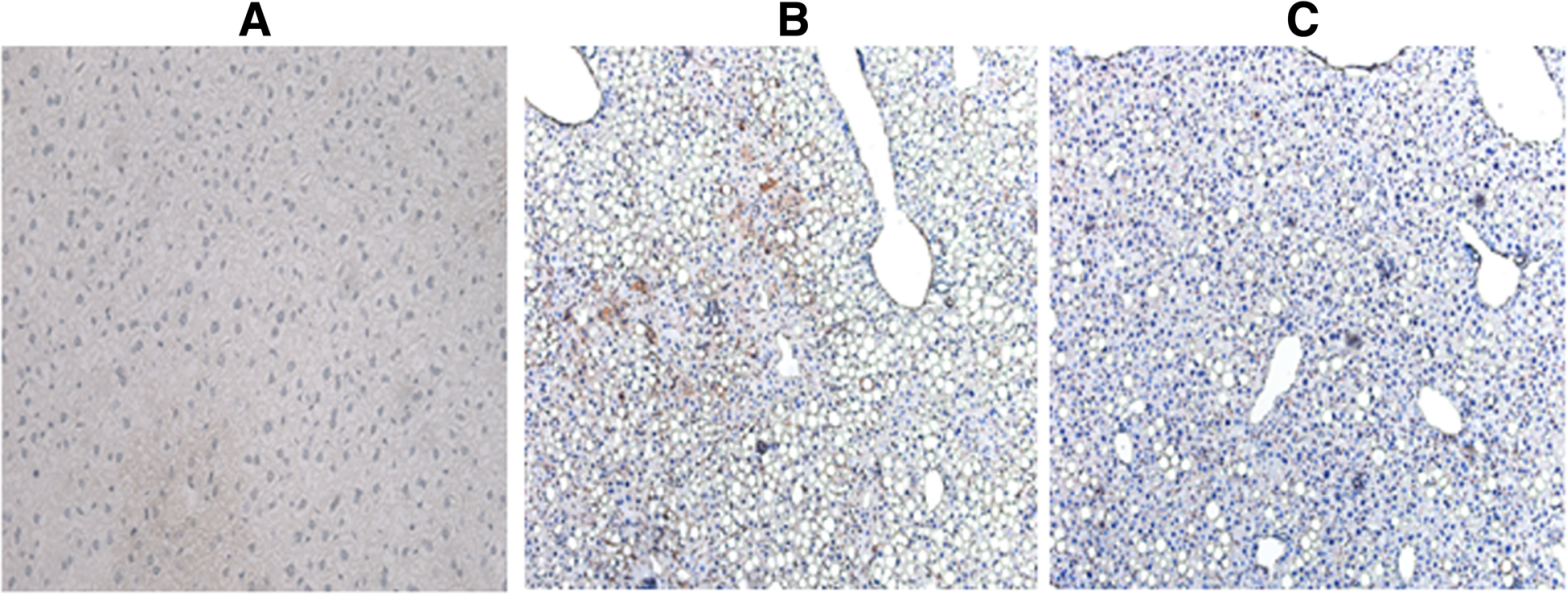
The *Alisma* and *Rhizoma* decoction suppresses α-SMA expression in the hepatic tissues of mice with MCD-induced NASH. The liver tissues of mice receiving regular diet (**a**), MCD (**b**) and MCD plus the *A. oriental - R. atractylodis macrocephalae* decoction (**c**) were examined by immunohistochemistry for α-SMA. Brown-stained cells are α-SMA positive. Magnification, 100 ×

We next examined the effect of the *Alisma* and *Rhizoma* decoction on HSC in hepatic tissues. Immunohistochemistry revealed increased expression of α-SMA, a marker of activated hepatic stellate cells (HSCs), in the hepatic lobule and the sinusoidal space in group II versus group I (Fig. 4), suggesting HSC activation in the liver tissues of mice with MCD-induced NASH-like changes. In group III, α-SMA was only expressed scantly in the central vein and portal area, indicating that the *Alisma* and *Rhizoma* decoction suppressed α-SMA expression in the hepatic tissues of mice with MCD-induced NASH.

### The *Alisma* and *Rhizoma* decoction inhibits autophagy in hepatocytes in mice with MCD-induced NASH

**Fig. 5:**
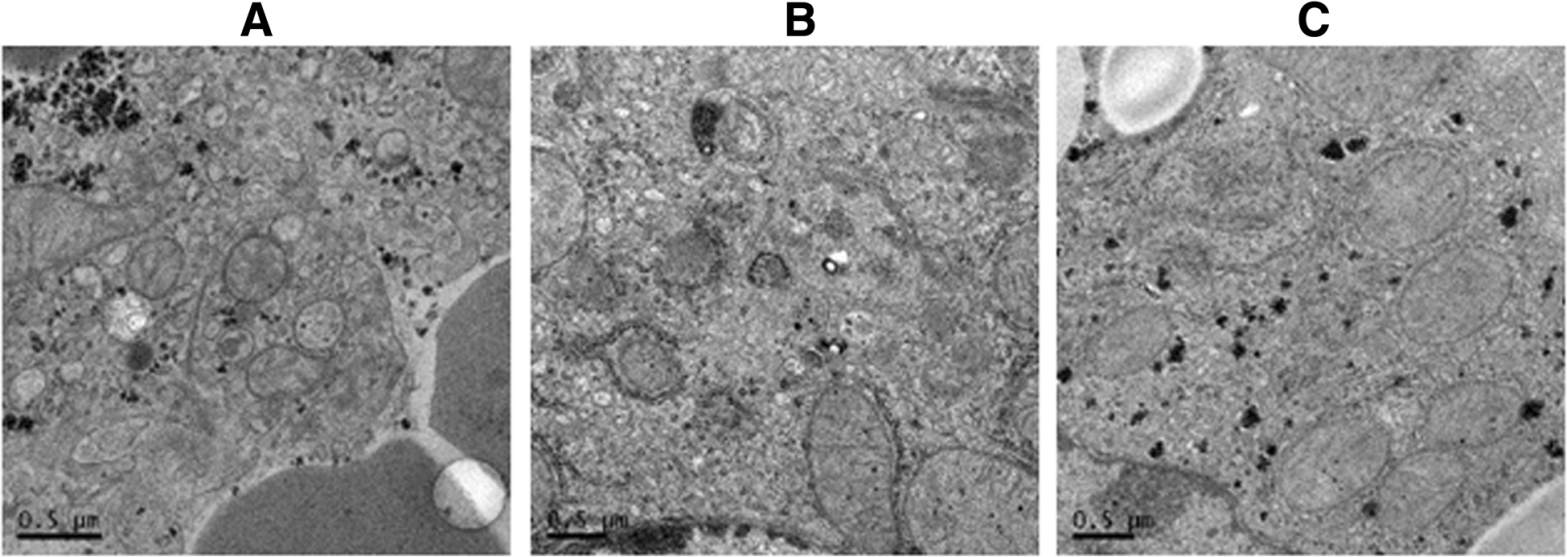
The *Alisma* and *Rhizoma* decoction inhibits autophagy in hepatocytes in mice with MCD-induced NASH. Transmission electronic microscopy reveals no autophagosomes in the control mice (**a**) while autophagosomes with bilaminar membranes are present inside the cells and some autophagosomes were fused with lysosomes and formed autophagolysosomes with monolayer membrane in group II (**b**). Treatment with the *Alisma* decoction markedly reduces the number of autophagosomes (**c**)

Recently, autophagy has been implicated in the development and progression of hepatic fibrosis [5–7]. Our transmission electronic microscopy revealed no autophagosomes in the control mice (Fig. 5a). In group II, autophagosomes with bilaminar membranes were present inside the cells and some autophagosomes were fused with lysosomes and formed autophagolysosomes with monolayer membrane (Fig. 5b), suggesting that autophagy was involved in NASH-induced liver injuries. Treatment with the *Alisma* and *Rhizoma* decoction markedly reduced the number of autophagosomes, indicating that the decoction may suppress NASH-induced liver fibrosis via inhibition of autophagy (Fig. 5c).

**Fig. 6:**
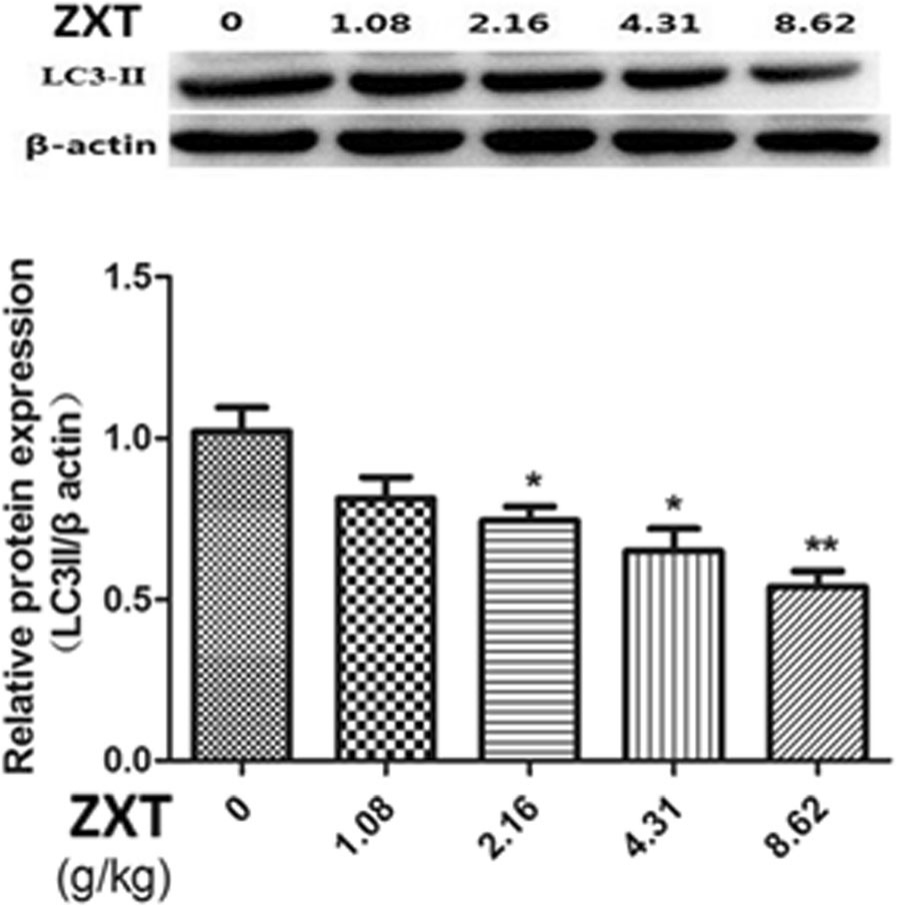
Immunoblotting assays show that the *Alisma* and *Rhizoma* decoction caused a dose-dependent decrease in LC3-IIin hepatic tissues. *P < 0.05, **P < 0.01

We additionally examined the expression of LC3-II, which correlates with the content of autophagosomes and reflects the activity of autophagosomes. Our immunoblotting assays showed that the *Alisma* decoction reduced LC3-II expression in hepatic tissues (Fig. 6, the full, uncropped western blots were supplied in the Additional files 1 and 2), suggesting that the decoction reduced the levels of autophagy in hepatic tissues.

## Discussion

The current study demonstrated that the *Alisma* and *Rhizoma* decoction lessened NASH-induced liver injuries in mice, which was associated with reduced levels of oxidative stress biomarkers and inhibition of autophagy in hepatic tissues. Inhibition of lipogenesis and anti-inflammatory activities have been described for *A. orientale* and *R. atractylodis macrocephalae* as individual medicinal herb [10, 14]. Though the *Alisma* and *Rhizoma* decoction has been shown to possess a myriad of biological and clinical effects, its mechanisms of action have not been elucidated. Our study shed light on the actions of the *Alisma* and *Rhizoma* decoction by showing that, apart from inhibition of lipogenesis and anti-inflammatory activities, the *Alisma* and *Rhizoma* decoction could alleviate NASH-induced liver injuries by suppressing oxidative stress and autophagy in hepatocytes.

The mechanisms underlying the pathogenesis of NAFLD have not been fully elucidated. It has been hypothesized that fatty liver degeneration as a result of intracellular triglyceride accumulation in hepatocytes represents the primary hit while subsequent oxidative stress and inflammatory response are second hits [27, 28]. Numerous studies have shown that NASH occurs as a result of oxidative stress and mitochondrial injury, which correlates with severity of inflammation in NASH [29–32]. Therapy aiming at oxidative stress may lessen liver inflammation and fibrosis in NASH [33]. In vitro findings indicate that *A. orientale* attenuated long-chain saturated fatty acid-induced cellular injury by inhibiting oxidative stress [26]. We also showed in this study that the *Alisma* and *Rhizoma* decoction significantly accentuated the rise in intrahepatocellular SOD activities and suppressed MCD-induced increase in intrahepatocellular MDA contents, suggesting that the *Alisma* and *Rhizoma* decoction may alleviate NASH by inhibiting oxidative stress in the liver. In addition, drug delivery and kinetic of phytochemicals in the *Alisma* and *Rhizoma* decoction may play an essential role in their therapeutic effects in the liver [34].

Oxidative stress and its relevant pathway have a crucial role in liver injury and NAFLD [35]. Oxidative stress may initiate the cascade of HSC activation via the actions of reactive oxidative species (ROS) and lipid peroxidation product such as MDA [36], and activated HSCs are the main culprit of liver fibrosis. We found that the *Alisma* and *Rhizoma* decoction downregulated the expression of α-SMA, a marker of HSC activation, in hepatic tissues, suggesting that the *Alisma* and *Rhizoma* decoction could suppress HSC activation. Autophagy has been shown to be a regulator of lipid metabolism and lipophagy has been proposed to describe the role of autophagy in regulating hepatic lipid metabolism [37]. Recently, it has been shown that autophagy may promote activation and proliferation of HSCs [8, 9, 38, 39]. LC3 is a marker of autophagy and LC3-II reflects the activity of autophagy [40]. The current study showed that the *Alisma* and *Rhizoma* decoction downregulated the expression of LC3-II in hepatic tissues and reduced the number of autophagosomes, indicating that the *Alisma* and *Rhizoma* decoction could inhibit NASH by modulating autophagy.

## Conclusion

In summary, our study has shown that the *Alisma* and *Rhizoma* decoction lessens NASH-associated liver injuries by modulating oxidative stress and autophagy in hepatocytes of mice fed with MCD, suggesting that the *Alisma* and *Rhizoma* decoction be further explored for its therapeutic action in NAFLD. However, further studies are still required to identify its bioactive constituents, and elucidate the structure-activity relationship and detailed mechanisms of action of the *Alisma* and *Rhizoma* decoction.

## Additional files

Additional file 1: The full, uncropped western blots of LC3-II. (TIF 300 kb)

Additional file 2: The full, uncropped western blots of β-actin. (TIF 386 kb)

## Abbreviations

ALT: Alanine aminotransferase
ER: Endoplasmic reticulum
HSC: Hepatic stellate cells
MCD: Methionine and choline diet
NAFLD: Non-alcoholic fatty liver disease
NASH: Non-alcoholic steatohepatitis
