# Supplementary figures and images for: The Alisma and Rhizoma decoction abates nonalcoholic steatohepatitis-associated liver injuries in mice by modulating oxidative stress and autophagy
Source: BMC Complement Altern Med. 2019 Apr 29;19:92. doi: 10.1186/s12906-019-2488-6 (PMC6489313; doi:10.1186/s12906-019-2488-6)

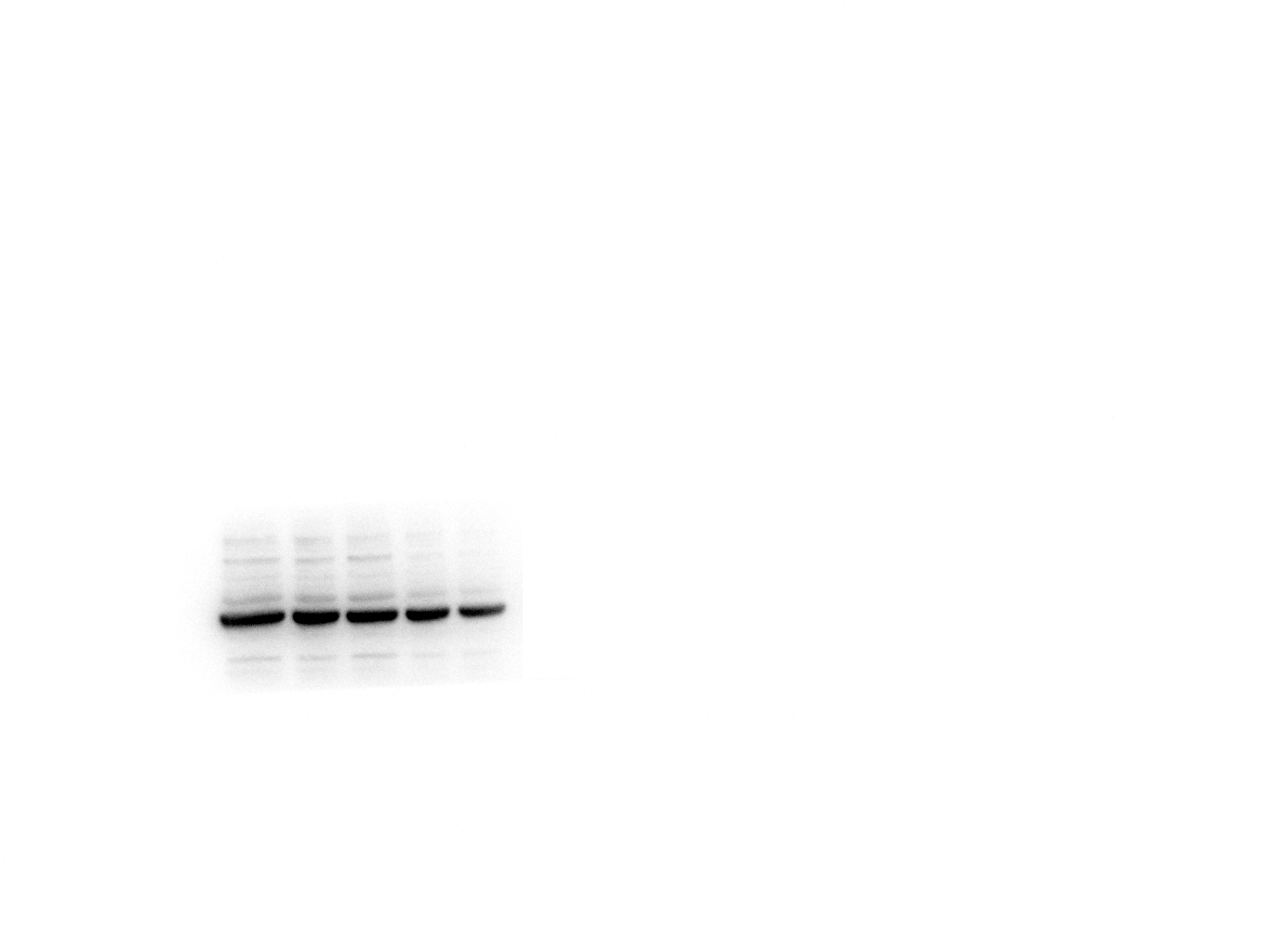

Supplement: Supplementary file 1 — The full, uncropped western blots of LC3-II. (TIF 300 kb) [file 12906_2019_2488_MOESM1_ESM.tif]

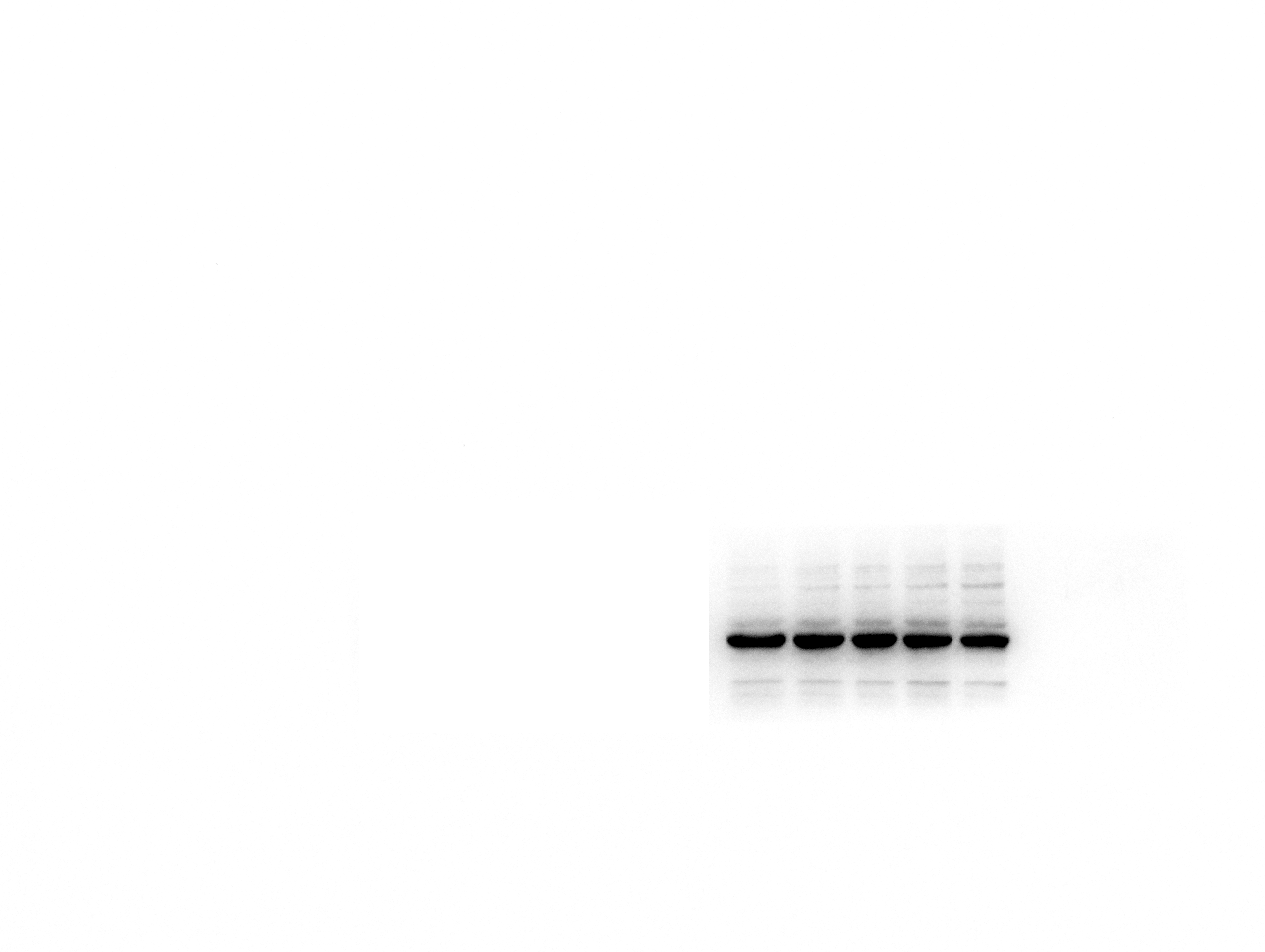

Supplement: Supplementary file 2 — The full, uncropped western blots of β-actin. (TIF 386 kb) [file 12906_2019_2488_MOESM2_ESM.tif]
